# The complete chloroplast genome sequence of *Aconitum tschangbaischanense* (Ranunculaceae)

**DOI:** 10.1080/23802359.2023.2220435

**Published:** 2023-06-08

**Authors:** Xuelian Liu, Junyi Zhu, Mingge Jiang, Shengchao Guan, Liqiu Zhang, Haiying Zhao

**Affiliations:** a College of Life Science, Tonghua Normal University, Tonghua, China; b Key Laboratory of Evaluation and Application of Changbai Mountain Biological Germplasm Resources of Jilin Province, Tonghua, China; c College of Medical, Tonghua Normal University, Tonghua, China; d College of History and Geography, Tonghua Normal University, Tonghua, China

**Keywords:** *Aconitum tschangbaischanense* S. H. Li & Y. H. Huang, 1975, chloroplast genome, endangered species

## Abstract

The perennial herbal medicine species *Aconitum tschangbaischanense*, is endemic to Changhai Mountain, Jilin province. In this study, we attempted to uncover the complete chloroplast (cp) genome of *A. tschangbaischanense* based on sequencing data using the Illumina sequencing technology. As per the results: (1) the length of its complete cp genome is 155,881 bp with a typical tetrad structure; (2) the structure of its cp genome contains large single-copy and small single-copy (LSC and SSC) regions of 86,351 and 16,9444 bp, respectively, isolated by two inverted repeat regions (IRs) of 26,293 bp; (3) we annotated a total 131 genes, consisting of 86 protein-coding genes, eight rRNA genes, and 37 tRNA genes. According to the maximum-likelihood phylogenetic tree based on complete cp genomes, *A. tschangbaischanense*, showed close association with *A. carmichaelii*, which belongs to clade I. Finally, this study provides the characteristics of the cp genome of *A. tschangbaischanense*, and its phylogenetic position.

*Aconitum* (Ranunculaceae) is a perennial genus that includes more than 400 species and is mainly distributed in the temperate regions of the Northern Hemisphere (Li and Kadota 2001). China is one of the diversity centers for *Aconitum* with more than 200 species, and approximately 76 species are utilized in traditional Chinese medicine (Li and Kadota 2001; Xiao et al. 2006). *Aconitum tschangbaischanense* S. H. Li & Y. H. Huang, 1975, is an endemic species limited to the Changbai Mountain area between 1700 and 2100 m (Zhou 2009) (Figure 1). It is only concentrated in the meadows of the *Betula ermanii* belt and alpine tundra belt in the Changbai Mountain National Nature Reserve. Species with narrow distributions are at risk of extinction. It is listed as an endangered species and is in urgent need of protection (Zhou 2006); however, it has not been assessed by the International Union for Conservation of Nature (IUCN). Therefore, it is necessary to study its genetic diversity to guide its *ex situ* and *in situ* conservation. We assembled the complete plastome of *A. tschangbaischanense* to (1) characterize and compare the complete plastome of *A. tschangbaischanense* and (2) assess the taxonomic position of *A. tschangbaischanense*.

**Figure 1.**
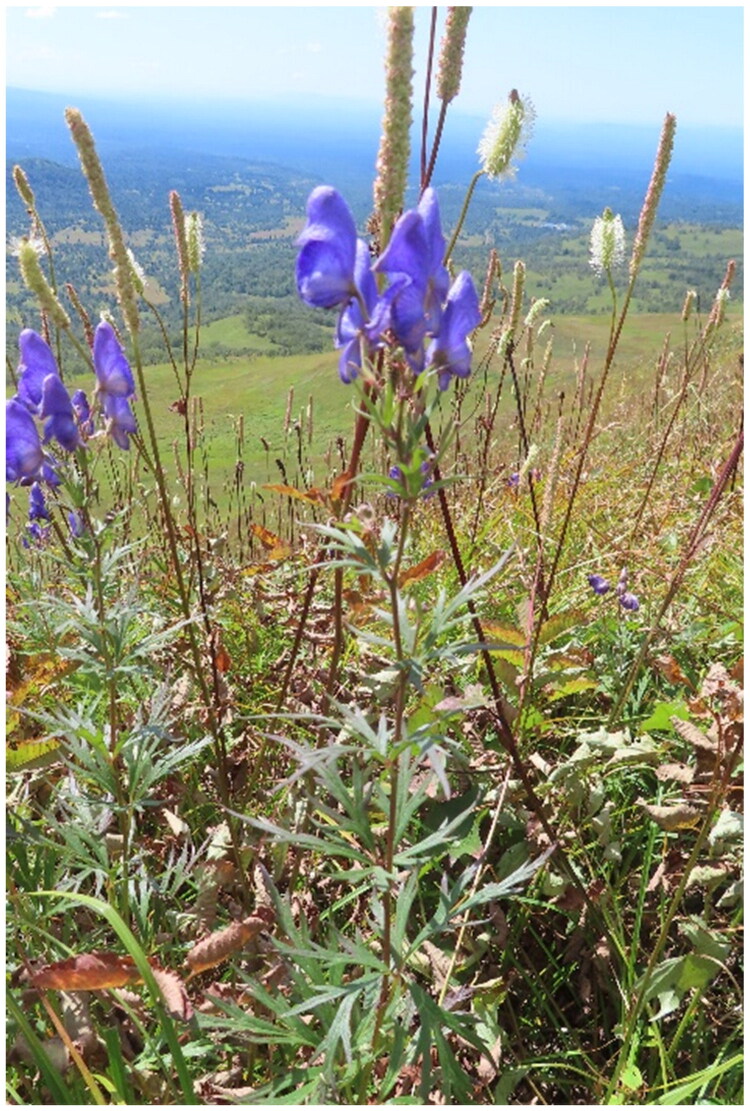
Species reference image of *A. tschangbaischanense*. The image was taken by Shengchao Guan.

We collected samples from the south slope of Changbai Mountain in Jilin province, China (128°2′E, 41°56′N, Alt.: 1850 m). This work was approved by the Changbai Mountain Nature Conservation Management Center and complies with the IUCN policy research involving species at risk of extinction (see Guidelines for appropriate uses of IUCN Red List Data), the Convention on Biological Diversity and the Convention on the Trade in Endangered Species of Wild Fauna and Flora. The silica gel dried leaves and specimens were deposited at the herbarium of Tonghua Normal University (Xue-Lian Liu, liuxuelian1023@163.com) under the voucher number LXL202115 (plant identification was completed by Professor You Zhou of Tonghua Normal University). Total genomic DNA was extracted from the dried leaves using the CTAB method (Doyle and Doyle 1987). Paired-end sequencing of whole sequences from both ends of 150 bp fragments was performed on the DNBSEQ T7 at Benagen (https://www.benagen.com), and approximately 4 Gb clean data were generated for *A. tschangbaischanense.* A total of 28,270,584 clean reads and 4,228,588,412 clean bases were produced for *de novo* assembly using the GetOrganelle pipeline (Jin et al. 2020). *Aconitum kusnezoffii* was used as a reference for annotation using the program CPGAVAS2 (Shi et al. 2019). We also used CPGview (http://www.1kmpg.cn/cpgview) to improve annotation (Liu et al. 2023). To identify the phylogenetic position of *A. tschangbaischanense*, the maximum-likelihood (ML) tree was reconstructed based on 40 species of complete chloroplast (cp) genomes using IQtree V2.1.3.

The genome sequence data that support the findings of this study are openly available in GenBank of NCBI at https://www.ncbi.nlm.nih.gov under accession no. OP221050. The read coverage depth map is shown in Figure S1. The complete cp genome of *A. tschangbaischanense* has a quadripartite structure comprising 155,881 bp in length, with 38% of the overall guanine and cytosine (GC) content. There is a LSC region of 86,351 bp and an SSC region of 16,944 bp, which are separated by a pair of inverted repeat (IR) regions (IRa and IRb) of 26,293 bp in length. The GC contents of the corresponding values in the LSC, SSC, and IR regions were found to be 38.14%, 31.64%, and 45.23%, respectively (Figure 2).

**Figure 2.**
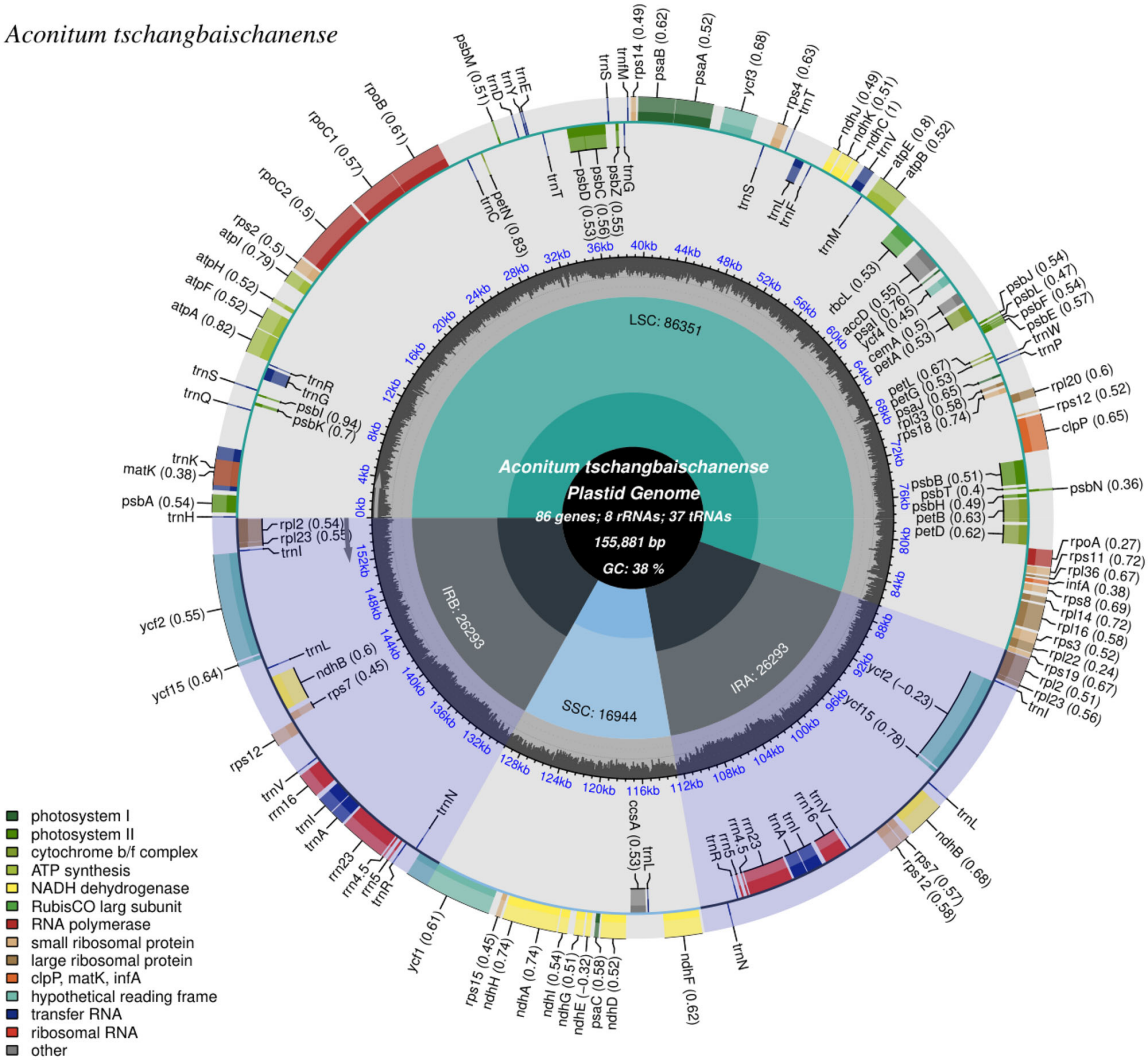
The map of *A. tschangbaischanense* chloroplast (cp) genome by CPGview. The map contains the core area and two tracks. The basic information of the cp genome shows in the core area. The GC content along the genome is plotted on the inner track. The genes showed on the external track, and genes with different functional groups are identified by different colors.

A total of 131 predicted genes in the cp genome of *A. tschangbaischanense* were assigned to three groups based on their functions: 86 protein-coding genes, 37 tRNA genes, and eight rRNA genes (Figure 2). Twelve cis-splicing gene were identified. Compared to *Arabidopsis thaliana*, the rps16 gene was not generated in the cp genome of *A. tschangbaischanense* (Liu et al. 2023; Figure 3(a)). The structure of trans-splicing gene *rps*12 of *A. tschangbaischanense* showed there are three unique exons. Two of them are duplicated as they are located in the IR regions (Figure 3(b)). We also identified 35 simple sequence repeats, 32 long tandem repeats, and 19 long tandem repeats. The number of mono-, di-, tri-, and tetra-nucleotides SSRs was 22, 11, 1, and 1, respectively.

**Figure 3.**
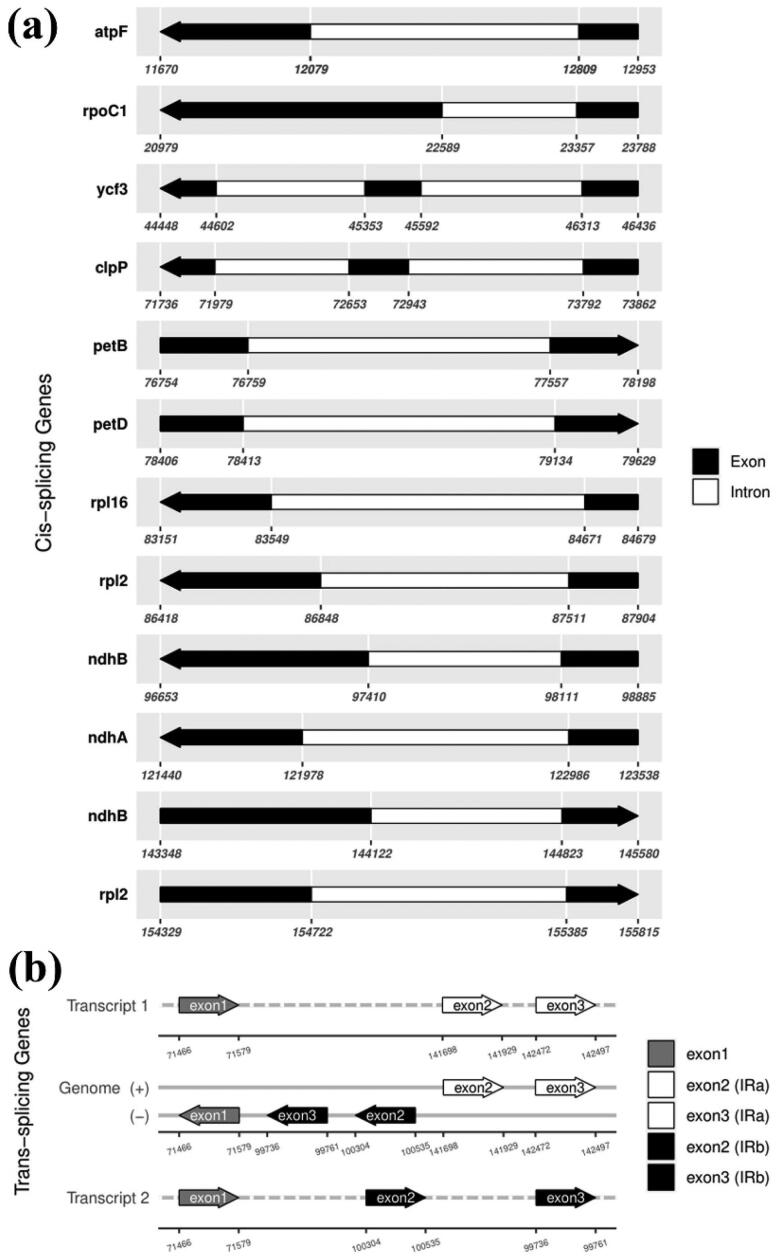
Schematic of the cis-splicing gene map (a) and trans-splicing genes map (b) generated for the chloroplast genome of *A. tschangbaischanense*. (a) The genes are arranged from left to right based on their order on the chloroplast genome. The gene names are shown on the top, and the gene structures are on the bottom. The exons are shown in black; the introns are shown in white. The arrow indicates the sense direction of the gene. Please note that lengths of exons and introns are not drawn to scale. (b) The start and end positions on the pre-mRNA are shown below the line. The lines represent the genome plus (+) and minus (−) DNA strands. The arrowheads represent the corresponding exons of the rps12 genes.

To understand the phylogenetic relationship of *A. tschangbaischanense* with related taxa in *Aconitum*, a ML tree was constructed using IQtree V2.1.3, based on the complete cp genomes of 40 Aconitum species and two outgroup species (*Gymnospermium microrrhynchum* and *Delphinium grandiflorum*) (Chen et al. 2015; Choi et al. 2016; Duan et al. 2019; Kim et al. 2022; Kong et al. 2017; Kong et al. 2018; Li et al. 2020; Lim et al. 2017; Lim et al. 2020; Liu et al. 2020; Ni et al. 2022; Park et al. 2017; Wang and Li 2020; Xia et al. 2022; Yang et al. 2022; Zhang et al. 2021). The ML tree showed that all the species in our study were divided into six main clades, belonging to two subgenera (subgenus *Aconitum* and *Paraconitum*). There are two clades with only one species, *A. tanguticum* and *A. coreanum*. Ten, seven, and 11 species were grouped into clades I, II, and III, respectively, which belonged to the subgenus *Aconitum*. Clade VI has 12 species and formed the subgenus *Paraconitum*. The plastid phylogenomic tree indicated that *A. tschangbaischanense* was closely related to *A. carmichaelii*, which belongs to the subgenus Aconitum (Figure 4).

**Figure 4.**
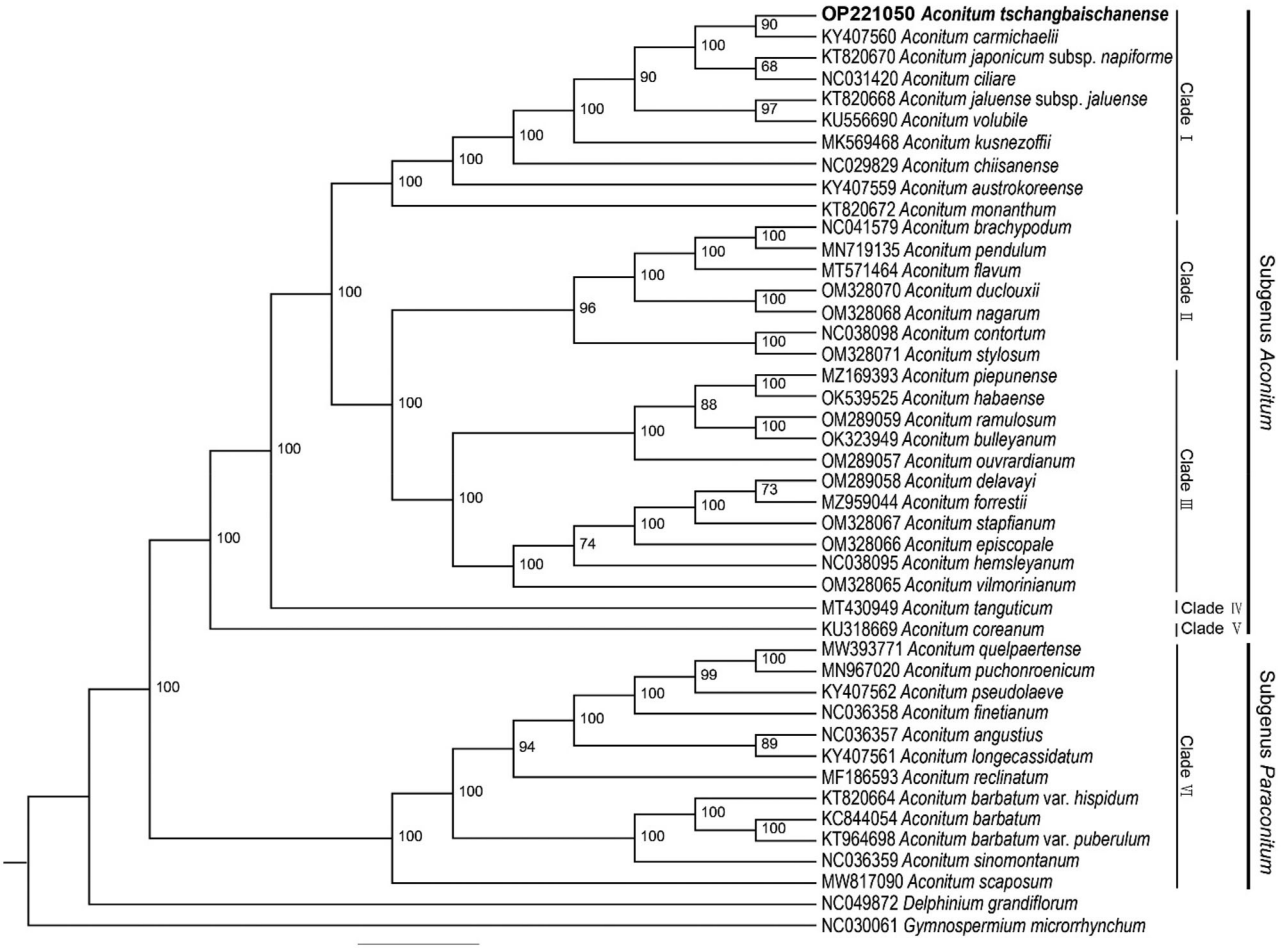
Maximum-likelihood (ML) tree based on complete chloroplast genome of 42 taxa including *A. tschangbaischanense.* We directly listed the cited publications used in the phylogenetic tree in the references part.

## Supplementary Material

Supplemental Material

## Data Availability

The genome sequence data that support the findings of this study are openly available in GenBank (https://www.ncbi.nlm.nih.gov/) under accession no. OP221050. The associated BioProject, SRA, and Bio-Sample numbers are PRJNA906719, SRR22459652, and SAMN31936196, respectively.
