# Supplementary material for: The complete chloroplast genome sequence of Aconitum tschangbaischanense (Ranunculaceae)
Source: Mitochondrial DNA B Resour. 2023 Jun 8;8(6):658–62. doi: 10.1080/23802359.2023.2220435 (PMC10251779; doi:10.1080/23802359.2023.2220435)
Supplement: Supplemental Material [file TMDN_A_2220435_SM2537.docx]

Figure S1 The read coverage depth map of *A. tschangbaichanense*.
